# Individual and social vulnerabilities upon acquiring tuberculosis: a literature systematic review

**DOI:** 10.1186/1755-7682-7-35

**Published:** 2014-07-12

**Authors:** Sheylla Nadjane Batista Lacerda, Rayrla Cristina de Abreu Temoteo, Tânia Maria Ribeiro Monteiro de Figueiredo, Fernanda Darliane Tavares de Luna, Milena Alves Nunes de Sousa, Luiz Carlos de Abreu, Fernando Luiz Affonso Fonseca

**Affiliations:** 1 Faculdade de Medicina do ABC, Santo André, SP, Brazil; 2 Universidade Estadual da Paraíba, Paraíba, PB, Brazil; 3 Universidade de São Paulo, São Paulo, SP, Brazil; 4 Universidade de Franca, Franca, SP, Brazil

**Keywords:** Tuberculosis, Vulnerability, Social vulnerability, Vulnerability analysis, Vulnerability study, Health vulnerability, Transmissible diseases prevention

## Abstract

Tuberculosis is a contagious infectious disease mainly caused by the bacteria *Mycobacterium tuberculosis* that still meets the priority criteria - high magnitude, transcendence and vulnerability - due to the threat it poses to public health. When taking into consideration the vulnerability conditions that favor the onset of the disease, this article aimed to investigate the implications originated from individual and social vulnerability conditions in which tuberculosis patients are inserted. Databases like MEDLINE, LILACS and SciELO were searched in Portuguese, Spanish and English using the descriptors tuberculosis and vulnerability, and 183 articles were found. After the selection criterion was applied, there were 22 publications left to be discussed. Some of the aspects that characterize the vulnerability to tuberculosis are: low-income and low-education families, age, poor living conditions, chemical dependency, pre-existing conditions/aggravations like diabetes mellitus and malnutrition, indigenous communities, variables related to health professionals, intense border crossings and migration, difficulty in accessing information and health services and lack of knowledge on tuberculosis. Much as such aspects are present and favor the onset of the disease, several reports show high incidence rates of tuberculosis in low vulnerability places, suggesting that some factors related to the disease are still unclear. In conclusion, health promotion is important in order to disfavor such conditions or factors of vulnerability to tuberculosis, making them a primary target in the public health planning process and disease control.

## Resumo (PORTUGUÊS)

A tuberculose é uma doença infectocontagiosa, causada principalmente pela bactéria *Mycobacterium tuberculosis*, que ainda atende aos critérios de priorização de um agravo em saúde pública, os quais: alta magnitude, transcendência e vulnerabilidade. Levando-se em consideração as condições sociais e individuais de vulnerabilidade que favorecem ao adoecimento por TB, o artigo teve o objetivo de investigar as implicações provenientes das condições de vulnerabilidades que estão inseridas as pessoas que adoecem de tuberculose. Em busca realizada às bases de dados Medline, Lilacs e Scielo, utilizando os descritores tuberculose e vulnerabilidade (também nas línguas inglesa e espanhola), foram encontrados 183 artigos, após serem aplicados critérios de seleção, restaram 22 publicações a serem discutidas. Dentre os aspectos elencados que caracterizam a vulnerabilidade ao adoecimento por TB: baixos níveis de renda e de escolaridade; idade; condições relacionadas à moradia; dependência química; condições/agravos preexistentes (diabetes mellitus, desnutrição); indígenas; variáveis relacionadas ao trabalho na área da saúde; fluxo intenso transfronteiriço e migração; dificuldade de acesso às informações e à saúde; falta de conhecimento sobre a TB. Por mais que estes aspectos estejam presentes e favoreçam o adoecimento, existem relatos de altas taxas de incidência de tuberculose, mesmo em localidades com baixa vulnerabilidade, o que sugere a existência de fatores relacionados à tuberculose ainda pouco esclarecidos. Contudo, é necessário promover saúde no sentido de desfavorecer tais condições ou fatores de vulnerabilidade à doença, tornando-os parte primordial do processo de planejamento da saúde e controle da tuberculose.

## Introduction

Tuberculosis (TB) is a contagious infectious disease, can be caused by the species complex bacteria as Mycobacterium *tuberculosis*[1]. It most commonly occurs in the lungs (pulmonary tuberculosis), but it can also attack other organs (extrapulmonary tuberculosis). Although it is a disease known for centuries, tuberculosis is still a major health concern given the fact it is airborne spread and it affects particularly immune-deficient patients and impoverished populations. Moreover, it meets the priority criteria of a public health aggravation: high magnitude, transcendence and vulnerability [2].

The concept of vulnerability is quite encompassing and implicit in multiple professional areas. It concerns people’s susceptibility or the probability of exposition to a disease which result from a group of aspects, both individual and collective, that include the context the individual is inserted in. These aspects express illness potentials (debilities/fragilities), disease hindrance and coping strategies (resistance and creative ability to overcome adverse scenarios) related to each and every individual. In addition, such concept also serves as an indicator of iniquity and social inequality. The concept of risk, on the other hand, is defined as the probability and chance of population groups to get sick and die owing to some health aggravation. Vulnerability precedes risk, and it establishes the different risks a person faces to get infected, get sick and die [3-5].

Owing to persistent and recrudescent TB events in many locations, the Directly Observed Treatment Short-course (DOTS) strategy was launched in 2001, trying to solve, or at least minimize, the problem. The biggest challenges to be faced, given the gravity of the disease, are the social inequality, the onset of Acquired Immunodeficiency Syndrome (AIDS), the population ageing and large migratory movements [6].

When it comes to diseases whose onset is strongly associated with socio-economic issues (TB as an example), it is paramount to discuss questions connected to vulnerability, as the relations established within the same social, economic, environmental and cultural contexts generate a higher or lower vulnerability to *M. tuberculosis* contamination. Therefore, the availability or unavailability of protection resources (financial means and access to health services, social support groups or information on the disease among others) interferes with the susceptibility to the disease [7].

In this research only the vulnerability expressed by the social and individual potential issues related to tuberculosis acquisition was taken into consideration. Based on the concept described above, the following guiding question was proposed: Which individual and social vulnerability context are people who get tuberculosis inserted in? Thus, the current article aimed to investigate the implications originated from the social and individual vulnerability conditions in which people who get tuberculosis are inserted.

## Methodology

Literature searches were performed using the databases Medical Literature Analysis and Retrieval System Online (MEDLINE) (http://www.ncbi.nih.gov/pubmed), Latin America and Caribbean Health Sciences (LILACS) (bases.bireme.br) and Scientific Electronic Library Online (SciELO) (http://www.scielo.org). The terms used in this systematic review were obtained from the Health Sciences Descriptors (DeCs) (decs.bvs.br) On the searches the combination of the descriptors tuberculosis and vulnerability in English was also used in Portuguese (*tuberculose* and *vulnerabilidade*) and Spanish (*tuberculosis* and *vulnerabilidad*) (Table 1).

**Table 1:** Amount of publications according to the descriptors from January, 2003 to May, 2013

**Databases**	***“Tuberculosis” “vulnerability”***	**“Tuberculose” “vulnerabilidade”**	***“Tuberculosis” “vulnerabilidad”***
MEDLINE	94	00	00
LILACS	18	20	14
SCIELO	15	10	12

Source: Database, 2013.

When the DeCS website was consulted using the descriptor *vulnerabilidade* in Portuguese the following possibilities came up: *análise de vulnerabilidade* (vulnerability analysis and *análisis de vulnerabilidad*), *vulnerabilidade social* (social vulnerability and *vulnerabilidad social*), *estudo sobre vulnerabilidade* (vulnerability study and *estudio de vulnerabilidad*) and *vulnerabilidade em saúde* (health vulnerability and *vulnerabilidad en salud*). Then the searches were performed with all the mentioned descriptors linked to *tuberculose* (tuberculosis), and the same articles from the previous searches came up. Therefore, search by using only the descriptor *vulnerabilidade* (vulnerability and *vulnerabilidad*) was the option taken.

No descriptor for vulnerability was found in the Medical Subject Headings (MeSH) database; however, some articles were found in the MEDLINE database when the descriptors mentioned above were used, and for this reason it was decided to perform the search on the Medline/PubMed database by using the same descriptors used on the other databases.

The search totaled 183 documents. Upon reading the titles, it was observed that 63 repeated themselves on the different databases, so 120 were left for analysis. Following the inclusion criteria, unconventional publications (consensuses, editorials, guidelines, evaluations and correspondences), project documents, clinical trials in laboratories, event summaries (congresses, conferences and seminars), literature reviews and articles that were not freely available in full text were disregarded. The search was also limited by time, and only publications from the past 10 years (from January 2003 to May 2013) were considered. In the end, by means of the reading of the titles and summaries, 89 articles were left for evaluation concerning the study objective.

All authors independently took part in the eligibility evaluation and the subsequent analysis of the publications. In the event of disagreement, the issue was consensually settled, and whenever questions concerning the summaries were raised, the documents were fully read.

### Screening of the articles

After reading the summary of 89 articles, 67 were excluded given the fact they were related to vulnerabilities of multidrug resistant patients with TB (MDR-TB patients), people with HIV/AIDS, malaria and other health implications that did not deal with TB cases. Therefore, 22 publications which suited the research objective were set aside.

All the articles showing results related to the vulnerabilities of TB patients or the aspects that characterize the vulnerability to TB were included.

A specific formulary was developed for data extraction. Information on the study title, country of origin, year of publication, author, language, type of study, objective and aspects that characterize the vulnerability to TB were entered in the formulary. The studies were classified according to study design, author, journal of publication, country, studied sample and the aspects that characterize the vulnerability to TB.

## Results

**Figure 1:**
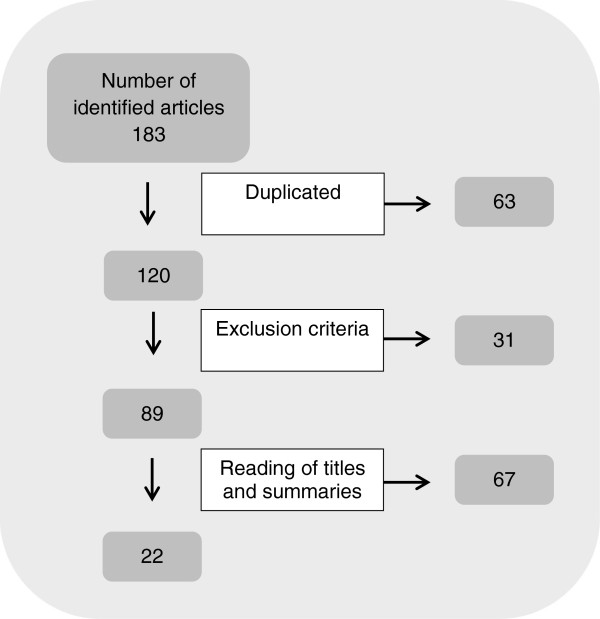
Flowchart showing the methodological pathway of the search, from January 2003 to May 2013.

A total of 22 articles were screened for discussion according to the established criteria for a bibliographic review (Figure 1).

Among the 22 studies that showed the aspects that characterized vulnerability to TB, five were classified as descriptive, five as transversal, two as case reports, two as qualitative, one as retrospective cohort, one as case control, one as ecological and one as prospective and retrospective (Table 2).

**Table 2:** Data screened from the articles inserted in the review from January 2003 to May 2013

**Ref.**	**Country**	**Year**	**Type of Study**	**Sample/Data aggregation**	**Aspects that characterize higher social and individual vulnerability to TB**
[8]	Brazil	2012	Ecological	Health administrative areas	Low income and educational levels
[25]	Brazil	2012	Descriptive (quantitative)	40 nurses and 36 nursing students	Lack of knowledge on the disease
[15]	Brazil	2011	Transversal	106 students	Age group 20–49 years, lack of knowledge on the disease
[10]	Brazil	2012	Transversal	Notified data on all TB patients	Low income level, difficulty accessing information and health services, intense border crossing, indigenous people
[9]	Brazil	2012	Descriptive case study	55 subjects (36 health professionals and 19 TB patients	Low income and educational levels, number of people per household
[13]	Brazil	2005	Descriptive retrospective	532 TB patients’ medical records	Elderly patients
[20]	Brazil	2005	Prospective and retrospective*	9 indigenous communities	Indigenous people
[38]	Chile	2012	Case control	Secondary data of 473 cases and 507 control	Diabetes Mellitus patients
[12]	South Africa	2010	Transversal	1,080 participants living in 336 dwellings (173 houses and 163 shanties)	Low income level (poor living conditions)
[30]	Tajikistan	2011	Transversal	509 migrating workers	Migrants
[18]	Índia	2011	Qualitative (Focal groups and interviews)	44 TB patients, 8 health professionals and 8 TB patients’ family members	Excessive alcohol intake
[17]	Pakistan	2012	Cohort retrospective	6,613 children under 15 in contact with TB patients	Contact with infected/bacilliferous people
[19]	Hungary	2009	Transversal	186 IV drug users	Use of IV drugs
[24]	Brazil	2011	Descriptive (qualitative)	19 TB patients	Lack of knowledge on the disease
[5]	Brazil	2010	Descriptive (exploratory)	26 TB patients	Low educational level, difficulty accessing information and health services, number of people sharing the same sleeping room
[11]	Brazil	2012	Descriptive case study	1 TB Control Program Team and 1 Family Health Team	Low income level (poor living conditions), malnutrition, unemployment or excessive working hours, excessive alcohol intake and use of other drugs
[22]	Brazil	2007	Descriptive	81 nursing workers	Lack of knowledge on the disease
[14]	Brazil	2013	Qualitative	7 TB patients	Elderly patients
[23]	Madrid	2009	Transversal	75 TB patients	Lack of knowledge on the disease
[21]	Europe	2006	Transversal	Representatives of 22 countries	Prison inmates
[16]	Washington	2006	Cohort	-	Homeless
[7]	Pakistan	2003	Transversal	386 prisoners	Prison inmates

*Transversal, cohort, documental, review - a large number of objectives were used, and many studies within the same research project were performed with different methodologies.

Source: Database 2013.

A number of aspects that characterize the social and individual vulnerability to TB were found: low-income [7-12] and low-educational levels [5,8,9]; variables related to age [13-15]; number of people per household [9]; number of people sharing the same sleeping room [5]; homeless people [16]; contact with bacilliferous patient [17]; alcoholism and/or use of other drugs [11,18,19]; preexisting conditions/implications (diabetes mellitus, malnutrition); populations living in special conditions, like indigenous communities [10,20]; and prison inmates [7,21]; variables related to health professionals (excessive working hours, unemployment) [11]; mobility (intense border crossings, migration) [10]; difficulty in accessing information and health services [5,10]; lack of knowledge on tuberculosis [15,22-25]. Such criteria used to characterize the vulnerability to the onset of the disease were differently developed and shown by the authors as seen in Table 2.

## Discussion

The analysis of the selected studies in the current bibliographic review showed that vulnerability is present in the TB context, thus confirming that TB is still a disease strongly related to socio- environmental and individual characteristics of a population.

In Ribeirão Preto, SP, a study revealed that the incidence of TB increased in areas where social vulnerability level was predominant. Through the Bayesian approach, the most significant covariate was considered for data representation due to the fact it had the lowest DIC (Deviance Information Criterion) value when compared with the other studied covariates (social vulnerability: DIC of 730.6; predominant family income level: DIC of 732.5; family educational level: DIC of 733.7), thus an indication of a closer relation between this covariate and TB incidence. However, in the year 2009 an expressive increase in the coefficients of TB incidence was observed, even in areas with no social vulnerability where income and educational levels were very high [8].

The main aspects that determine the role of poverty in the transmission of the *Mycobacterium tuberculosis* are: poverty’s influence on living conditions (overcrowded housing and poorly ventilated places), delayed diagnosis and increased vulnerability owing to malnutrition and/or HIV infection [26]. However, each social class comes across certain development conditions of productive forces and with specific social relations in which functioning and probabilities are established, a fact that may result in vulnerability effects for members from each social class [5,27].

The results obtained from Barbosa’s study [11] show that the poor living conditions (unhealthy housing, overcrowded accommodations, malnutrition, unemployment) of the studied population were the major cause of the onset of TB. A study conducted by Fasca [28] showed that poverty by itself does not determine the onset of TB. In areas where there is an outbreak of the disease, however, poor living conditions can be observed. Moreover, it is still a prevalent disease in poor urban areas where the transmission conditions are favored by high rates of contact and overcrowding.

A recent study performed in a low-cost housing complex identified people with tuberculosis. However, no anti-TB drugs were found, and none of the reported cases had been to any kind of health center in the previous 2 weeks, a fact that brings not only serious implications for the disease control, but also risks for other people who share the same residence [12].

The previous contact with a bacilliferous patient was considered as one more characterizing aspect of vulnerability to tuberculosis in this study. BATRA et al. [17] reported that mothers with TB play a considerable role in transmitting the disease to their children, female ones in particular. The authors explain that this may be due to the fact children spend more time closer to their mothers, leading to the reevaluation of the hypothesis that children do not play a significant part in the transmission of TB.

Besides the contact with a bacilliferous patient, the number of people per household has a direct relation to TB [29]. In residences with 4 or more people the risk of developing the disease is three-fold higher than in residences with two or less people [9]. Concerning the number of people sharing the same sleeping room, the higher this number is, the higher the risk of acquiring TB [1].

Vendramini [27], corroborating with the literature findings, observed a higher incidence of TB in social groups with lower educational levels. In the study of Gutiérrez [9], the population in question had from 4 to 7 years of education. When it came to educational level, most of the sample individuals from the study of Bowkalowski & Bertolozzi [5] did not complete the basic education program. Low educational levels may interfere with access to information in a broader sense, or more specifically, with the acquisition of information on the disease. The fragility of health services (low resolution rates) aggravates the scenario [5,10].

Homeless populations are especially vulnerable to tuberculosis. A study in King Country, Washington, analyzed an outbreak of TB among homeless people that made use of many shelters in the city [16].

The lack of knowledge on TB or the misconceptions on its transmission increased the individual’s vulnerability to the disease [24]. Studies on vulnerability have highlighted the importance of access to communication processes in an attempt to reduce susceptibility to aggravations and health-adverse circumstances [15].

As to nursing professionals’ knowledge on TB, some misconceptions on the most basic issues of the disease could be observed. Such a fact not only affects the assistance provided to patients, but also puts those professionals’ health at stake given the improper application of the disease prevention methods [27]. Even worse is the way the information on the disease is obtained. Many professionals seek guidance from educational institutions, means of communication, family and friends instead of health institutions, which impacts on the disease promotion and prevention [15].

In a study on the knowledge on TB, based on the higher rates of wrong answers, the nursing professionals involved were considered more vulnerable to the disease than the students. The questions included disease transmission during treatment, absolute protection provided by a surgical mask, the use of gloves as a biosafety measure for active pulmonary TB cases, and diagnostic methods for active TB [25].

Immigrants, in turn, are more vulnerable to TB owing to the living and working conditions they face in the new country. Moreover, the access to public health services is restricted when the immigrant is undocumented, and the cost of private services is in general very high. The average knowledge of an immigrant about TB is low and misunderstandings are frequent. In Tajikistan, although TB drugs are supplied free of charge, diagnoses and additional treatments are not. Therefore, a significant financial expense is generated to patients and their families [30].

The most vulnerable age group to develop TB is 20–40, a range that represents an economically active share of the population [15]. In Brazil, this range is between 20 and 49 years of age [31]. The Brazilian Ministry of Health (MH), however, considers that the risk to acquire TB is higher in children below 2 years of age and in adults above 60. The latter group in particular presents peculiar aspects that make those individuals different from the younger population, and such aspects result in physiological alterations (immunological, biochemical, psychosocial and morphological) and a higher vulnerability to infections [1].

Until very recently the elderly population in Brazil was not considered vulnerable to TB. Nevertheless, given the demographic transition in the country (adult and elderly population growth), TB incidence rate has gradually been rising among people in older age groups. The study by Oliveira et al. [13] showed a high mortality rate in this age group, a fact that reflected the higher vulnerability to the disease among elderly people. In a different study by Oliveira et al. [14], the researchers do not consider elderly people vulnerable to TB. However, they belong to an age group most vulnerable to the disease.

A study conducted with prison inmates in Pakistan reported that the prevalence of TB in the incarcerated population was higher when compared with the estimate in the general population. Environmental conditions in prisons, like overcrowding and poor ventilation, along with the presence of highly contagious cases are factors that increase vulnerability to TB among inmates [7].

Indigenous communities in Brazil are particularly vulnerable to TB. Incidence rates may be almost 10 times higher when compared with the Brazilian population in general [32-34]. However, the causes for this higher vulnerability are still unclear, and they may be related to the fast social-cultural changes, the high prevalence of malnutrition and overall deficiencies in health services [20].

In a study conducted by Ferraz [35] it was confirmed that the indigenous community and the border residents presented a much higher relative risk for TB than the general population of Mato Grosso do Sul State, OR = 7.3 and 1.7 respectively. A study performed in Rondonia State revealed that there is also a discrepancy in the incidence rates when TB in indigenous and non-indigenous populations were analyzed [10,36].

Biolchi’s study [10] was conducted in a region with a large concentration of indigenous population occupying border areas in its majority. As a probable result, a higher incidence of TB in those communities was observed. Due to their vulnerability to the disease, treatment must be provided as a top priority [37].

In the same study the author reported a higher incidence of TB in border municipalities when compared with other municipalities away from that area. The causes are the intense transborder crossings, cultural and economic integrations and a higher demand for health services, which are used not only by Brazilians, but also by residents from neighboring countries, overloading and making the access to health services more difficult [10]. Such environment leads to extensive multiplication of TB bacilli [1].

Individuals with Diabetes Mellitus (DM) are also prone to acquire TB owing to the decrease in their immunological response. The clinical and epidemiological implications in such cases are so noteworthy that the implementation of a screening process among this growing share of the population is advisable in order to obtain an early diagnosis of TB [38]. Besides, Leung et al. [39] showed that uncontrolled DM also favors the onset of TB.

Excessive working hours without the proper care make some health professionals more vulnerable to TB. A long period of professional experience in hospitals and extended work shifts (over 12 hours of work, including a second job) are factors that make such professionals more vulnerable to TB. On the other hand, different population segments, who also work long hours and are financially able to provide more than the bare minimum of support, are much less likely to be infected [11]. Therefore, knowing how long health professionals spend at work is quite important, as this highlights the period of exposure to disease agents, including M. tuberculosis [22].

The results obtained from Barbosa’s study [11] show that alcohol intake and the use of other drugs by some analyzed individuals were identified as determinants of TB risk in that studied territory. TB patients with alcohol abuse issues recognized that the excessive intake of alcohol was one of the reasons for their vulnerability to TB [18].

Contagious infectious diseases are among the main health effects of drug use. The study evaluated the vulnerability to infections related to drugs, and the results showed a prevalence of TB and other infections, like herpes, chlamydia, syphilis and gonorrhea among the interviewees [19].

All these aspects that characterize vulnerability to TB infection must be taken into consideration by health professionals in their daily routine at Community Health Centers. Such professionals must bear in mind that health promotion is characterized by a group of interventions which target the permanent, or at least the long-lasting elimination of the disease and tackle its most basic causes [40]. Therefore, health promotion aims to hinder the conditions or factors that contribute to the disease vulnerabilities, and all the mentioned factors are part and parcel of the health planning process and TB control [11].

## Conclusion

Despite the fact TB has always been related to low income and educational levels and poor living conditions, the current study shows that significant rates of the disease are found in the opposite scenario. Thus, the existence of more complex relations between TB incidence and a wide range of still unclear environmental and intrinsic factors are suggested, a matter to be studied in future researches.

On the other hand, those populations with heightened individual and social vulnerability to TB, like the ones reported in this review, must be prioritized, with the implementation of policies and actions that not only focus on specific issues, but also on the general aspects related to TB infection.

Furthermore, TB-related educational practices performed not only by means of informative lectures to the public in general, but also through information on the daily contact with the infected population are still very little used. Health professionals view these practices as a turning point to overcome the limits of the disease and to exchange information on its determinants and the possible coping strategies, according to different life experiences and the conditions and environmental factors each population group has to face.

Another highlighted issue is the fact that since the disease is concentrated among vulnerable groups, the majority of the population has a false sensation of safety. These individuals do not feel they are exposed to or threatened by TB infection; therefore, they end up ignoring awareness messages and overlooking TB symptoms in themselves or in people who they share residence with.

The strategy used for the search in the databases allowed us to present a concrete and current situational picture of vulnerability to TB, whose aim is to help develop control policies focused on this socially excluded population in particular. Without the proper balance and partial neutralization of these conditions, this share of the population will keep being an easier target for TB infection for a long time.

Nevertheless, a differentiated approach is necessary regarding the development of an action plan for TB control. It is vital that this action point to the diversity, like racial matters, ethnicities, and TB/HIV co-infection, which were not included in this study due to the limitations established for the search.

## Competing interests

The authors declare that they have no competing interests.

## Authors’ contribution

SNBL, RCAT, FDTL and TMRMF participated in the acquisition of data and revision of the manuscript. SNBL, RCAT, FDTL, TMRMF, MNAS, LCA, and FLAF conceived the study, determined the design and interpreted the data. SNBL, RCAT, FDTL and TMRMF drafted the manuscript. All authors read and gave final approval for the version submitted for publication.
